# Supplementary material for: Polymorphisms in ERCC5 rs17655 and ERCC1 rs735482 Genes Associated with the Survival of Male Patients with Postoperative Oral Squamous Cell Carcinoma Treated with Adjuvant Concurrent Chemoradiotherapy
Source: J Clin Med. 2019 Jan 1;8(1):33. doi: 10.3390/jcm8010033 (PMC6351919; doi:10.3390/jcm8010033)
Supplement: Supplementary file 1 [file jcm-08-00033-s001.pdf]

A

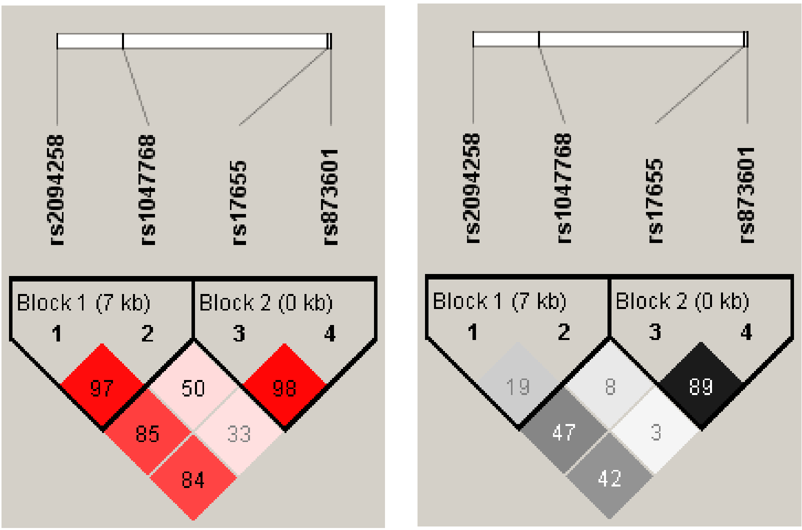

***ERCC5* (rs2094258, rs1047768, rs17655, and rs873601)**

B

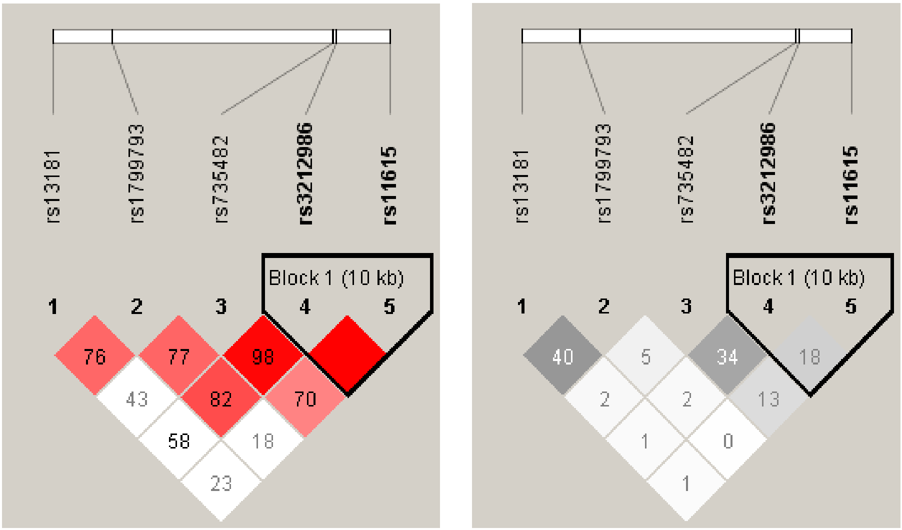

***ERCC2* (rs13181 and rs1799793) and *ERCC1* (rs735482, rs3212986, and rs11615)**

C

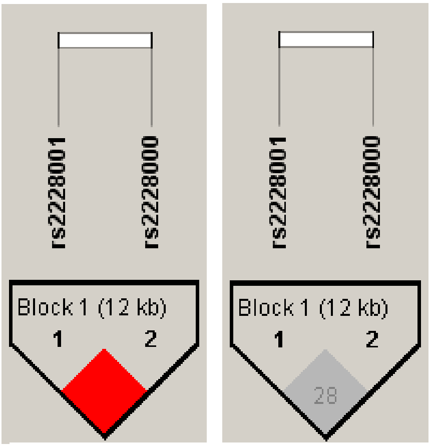

***XPC* (rs2228001 and rs2228000)**

D

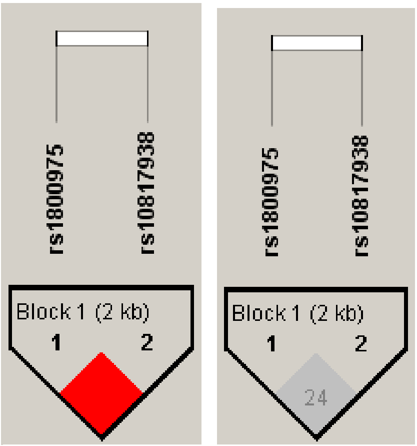

***XPA* (rs1800975 and rs10817938)**

Figure S1: Linkage disequilibrium (LD) analysis among single nucleotide polymorphisms (SNPs) within the same chromosome or gene. (A) LD block for *ERCC5* (rs2094258, rs1047768, rs17655, and rs873601), (B) LD block for *ERCC2* (rs13181 and rs1799793) and *ERCC1* (rs735482, rs3212986, and rs11615), (C) LD block for *XPC* (rs2228001 and rs2228000), and (D) LD block for *XPA* (rs1800975 and rs10817938). The numbers in squares indicate pairwise D' (left) or R^2 (right) values and corresponding shade of red or black represents the degree of LD between the polymorphisms.

Supplementary Material

Table S1: Frequency distributions of NER polymorphisms by clinopathological factors

	rs2094258	rs1047768	rs17655	rs873601	rs735482	rs3212986	rs11615	rs1799793	rs13181	rs2228001	rs2228000	rs1800975	rs10817938
Variable	GG/GA/AA	TT/TC/CC	GG/GC/CC	AA/AG/GG	AA/AC/CC	GG/GT/TT	CC/CT/TT	GG/GA/AA	TT/TG/GG	AA/AC/CC	CC/CT/TT	AA/AG/GG	TT/TC/CC
Tumor differentiation													
Well	20/21/8	25/22/4	17/21/13	15/23/13	19/23/9	24/22/5	23/23/5	44/7/0	40/11/0	16/30/5	25/23/3	17/28/6	31/19/0
Moderate	90/97/21	101/95/14	62/105/43	57/108/45	70/105/35	96/94/20	106/92/12	186/23/1	181/26/3	88/93/27	82/104/24	48/107/55	134/70/6
Poor	22/25/8	34/18/3	18/25/12	14/28/13	15/31/9	20/26/9	34/21/0	48/7/0	44/11/0	24/27/4	28/19/8	16/28/11	37/15/1
Unknown	1/2/0	2/1/0	1/2/0	1/2/0	0/2/1	2/1/0	1/2/0	3/0/0	2/1/0	1/2/0	2/1/0	2/1/0	2/1/0
p value§	0.962§	0.152§	0.694§	0.948§	0.310§	0.304§	0.039*§	0.739§	0.950§	0.260§	0.868§	0.757§	0.550§
Primary tumor size													
T1–T2	51/51/18	67/51/4	37/60/25	34/62/26	37/66/19	53/55/14	66/52/4	111/11/0	100/22/0	49/56/16	56/54/12	37/60/25	79/40/1
T3–T4	82/94/19	95/85/17	61/93/43	53/99/45	67/95/35	89/88/20	98/86/13	170/26/1	167/27/3	80/96/20	81/93/23	46/104/47	125/65/6
p value	0.320	0.137	0.935	0.947	0.594	0.915	0.396	0.181§	0.782§	0.699	0.679	0.372	0.485§
Nodal involvement													
N0–N1	40/46/13	51/46/5	31/47/24	28/49/25	29/50/23	52/37/13	55/42/5	87/14/1	83/18/1	48/39/15	39/48/15	31/53/18	69/32/0
N2–N3	93/99/24	111/90/16	67/106/44	59/112/46	75/111/31	90/106/21	109/96/12	194/23/0	184/31/2	81/113/21	98/99/20	52/111/54	135/73/7
p value	0.837	0.646	0.794	0.771	0.160	0.107	0.825	0.209§	0.472§	0.051	0.258	0.257	0.169§
Perineural invasion													
No	54/69/16	70/65/7	42/69/31	38/72/32	48/69/25	60/66/16	75/57/10	127/14/1	118/23/1	56/69/17	69/56/17	37/72/33	89/50/2
Yes	79/76/21	92/71/14	56/84/37	49/89/39	56/92/29	82/77/18	89/81/7	154/23/0	149/26/2	73/83/19	68/91/18	46/92/39	115/55/5
p value	0.500	0.413	0.922	0.982	0.835	0.764	0.353	0.650§	0.870	0.901	0.101	0.963	0.847§
Vascular invasion													
No	124/136/36	153/126/21	92/146/62	82/152/66	100/147/53	135/132/33	153/131/16	265/34/1	252/45/3	122/142/35	133/133/34	78/154/68	191/99/7
Yes	9/9/1	9/10/0	6/7/6	5/9/5	4/14/1	7/11/1	11/7/1	16/3/0	15/4/0	7/10/1	4/14/1	5/10/4	13/6/0
p value	0.651	0.397	0.467	0.907	0.100	0.452	0.836	0.635§	0.670§	0.667	0.045*	0.907§	0.775
Lymphatic invasion													
No	121/121/33	144/119/16	91/130/58	81/139/59	88/142/49	131/118/30	141/124/14	244/34/1	232/44/3	111/131/35	126/122/31	78/141/60	178/95/4

Yes	12/24/4	18/17/5	7/23/10	6/22/12	16/19/5	11/25/4	23/14/3	37/3/0	35/5/0	18/21/1	11/25/4	5/23/12	26/10/3
p value	0.159	0.255	0.153	0.139	0.502	0.046*	0.483	0.344§	0.426§	0.168	0.072	0.097	0.033*
Extranodal extension													
No	47/53/12	56/51/7	33/54/27	29/57/28	41/54/19	47/49/18	67/41/6	102/11/1	98/15/1	47/51/16	48/54/12	30/62/22	72/42/0
Yes	86/92/25	106/85/14	65/99/41	58/104/43	63/107/35	95/94/16	97/97/11	179/26/0	169/34/2	82/101/20	89/93/23	53/102/50	132/63/7
p value	0.895	0.847	0.719	0.723	0.619	0.084	0.132	0.746§	0.441§	0.468	0.939	0.562	0.837§
Pathologic TNM stage													
III	15/20/6	19/22/1	12/19/11	11/20/11	10/26/6	22/17/3	20/20/2	35/7/0	33/9/0	21/13/8	17/18/7	15/20/7	29/12/0
IV	118/125/31	143/114/20	86/134/57	76/141/60	94/135/48	120/126/31	144/118/15	246/30/1	234/40/3	108/139/28	120/129/28	68/144/65	175/93/7
p value	0.682	0.263	0.709	0.805	0.273	0.487	0.828	0.360§	0.469§	0.039*	0.448	0.269	0.461
Disease free survival event													
No	81/87/22	99/84/11	60/101/33	55/104/35	63/102/29	84/87/23	100/85/9	171/22/1	161/30/3	82/85/27	79/94/21	52/96/46	130/59/2
Yes	52/58/15	63/52/10	38/52/35	32/57/36	41/59/25	58/56/11	64/53/8	110/15/0	106/19/0	47/67/9	58/53/14	31/68/26	74/46/5
p value	0.982	0.710	0.049*	0.077	0.453	0.660	0.787	0.924§	0.467§	0.083	0.553	0.684	0.052§

* $p < 0.05$

§ Mantel-Haenszel Chi-square test

Supplementary Material

Table S2: Univariate association between NER candidate SNPs and OSCC survival in CCRT treated patients

		Overall Survival			Disease-free Survival		
SNPs	No.	Event	HR (95% CI)	p-value	Event	HR (95% CI)	p-value
ERCC5/XPG							
rs2094258							
GG	133	35	1.00		52	1.00	
GA	145	45	1.10 (0.70 – 1.71)	0.687	58	1.01 (0.69 – 1.48)	0.947
AA	37	8	0.70 (0.33 – 1.52)	0.369	15	0.92 (0.52 – 1.63)	0.772
Additive model			0.92 (0.67 – 1.26)	0.609		0.97 (0.75 – 1.26)	0.842
Dominant model	182	53	0.99 (0.69 – 1.42)	0.961	73	0.67 (0.32 – 1.39)	0.279
Recessive model	37	8	0.67 (0.32 – 1.39)	0.279	15	0.91 (0.53 – 1.57)	0.739
rs1047768							
TT	162	43	1.00		63	1.00	
TC	136	41	1.10 (0.72 – 1.70)	0.655	52	0.96 (0.67 – 1.40)	0.848
CC	21	5	0.74 (0.29 – 1.87)	0.520	10	0.98 (0.50 – 1.92)	0.955
Additive model			0.97 (0.70 – 1.36)	0.874		0.98 (0.74 – 1.30)	0.882
Dominant model	157	46	1.05 (0.69 – 1.59)	0.832	62	0.97 (0.68 – 1.38)	0.853
Recessive model	21	5	0.70 (0.29 – 1.74)	0.444	10	1.00 (0.52 – 1.91)	0.995
rs17655							
GG	98	27	1.00		38	1.00	
GC	153	43	1.00 (0.62 – 1.63)	0.986	52	0.85 (0.55 – 1.29)	0.439
CC	68	19	0.92 (0.62 – 1.63)	0.771	35	1.27 (0.80 – 2.02)	0.310
Additive model			0.96 (0.72 – 1.28)	0.788		1.12 (0.87 – 1.43)	0.372
Dominant model	221	62	0.98 (0.62 – 1.53)	0.913	87	0.98 (0.67 – 1.44)	0.912
Recessive model	68	19	0.91 (0.55 – 1.52)	0.729	35	1.40 (0.94 – 2.08)	0.094
rs873601							
AA	87	26	1.00		32	1.00	
AG	161	42	0.81 (0.50 – 1.33)	0.402	57	0.86 (0.56 – 1.34)	0.508
GG	71	21	0.85 (0.48 – 1.52)	0.585	36	1.23 (0.76 – 1.99)	0.399
Additive model			0.92 (0.68 – 1.23)	0.566		1.12 (0.87 – 1.44)	0.401
Dominant model	232	63	0.97 (0.60 – 1.59)	0.917	93	1.35 (0.91 – 2.00)	0.131
Recessive model	71	21	0.82 (0.52 – 1.30)	0.408	36	0.98 (0.65 – 1.46)	0.906
ERCC1							
rs735482							
AA	104	35	1.00		41	1.00	
AC	161	41	0.75 (0.48 – 1.19)	0.223	59	0.84 (0.56 – 1.26)	0.397
CC	54	13	0.84 (0.44 – 1.59)	0.590	25	1.38 (0.83 – 2.27)	0.211
Additive model			0.87 (0.64 – 1.20)	0.392		1.12 (0.86 – 1.47)	0.397
Dominant model	215	54	0.77 (0.50 – 1.19)	0.238	84	0.95 (0.65 – 1.39)	0.806
Recessive model	54	13	0.99 (0.55 – 1.78)	0.963	25	1.53 (0.99 – 2.38)	0.058
rs3212986							

GG	142	36	1.00		58	1.00	
GT	143	45	1.30 (0.83 – 2.03)	0.247	56	1.00 (0.69 – 1.45)	1.000
TT	34	8	0.99 (0.46 – 2.13)	0.970	11	0.88 (0.46 – 1.68)	0.690
Additive model			1.09 (0.80 – 1.50)	0.586		0.96 (0.73 – 1.26)	0.775
Recessive model	177	53	1.24 (0.81 – 1.91)	0.326	67	0.98 (0.68 – 1.40)	0.900
Dominant model	34	8	0.86 (0.41 – 1.77)	0.674	11	0.88 (0.47 – 1.63)	0.676
rs11615							
CC	164	39	1.00		64	1.00	
CT	138	45	1.21 (0.78 – 1.86)	0.397	53	0.87 (0.60 – 1.26)	0.460
TT	17	5	0.97 (0.38 – 2.47)	0.952	8	0.92 (0.42 – 2.00)	0.824
Additive model			1.09 (0.78 – 1.53)	0.608		0.91 (0.67 – 1.23)	0.528
Dominant model	155	50	1.18 (0.77 – 1.79)	0.450	61	0.88 (0.61 – 1.25)	0.464
Recessive model	17	5	0.88 (0.36 – 2.18)	0.790	8	0.98 (0.46 – 2.10)	0.952
ERCC2/XPD							
rs1799793							
GG	281	77	1.00		110	1.00	
GA	37	12	1.13 (0.61 – 2.10)	0.701	15	0.99 (0.58 – 1.71)	0.984
AA	1	0	–	0.988	0	–	–
Additive model			1.12 (0.60 – 2.06)	0.727		0.99 (0.58 – 1.71)	0.984
Dominant model	38	12	1.12 (0.61 – 2.09)	0.713	15	0.99 (0.58 – 1.71)	0.984
Recessive model	1	0	–	0.988	0	–	–
rs13181							
TT	267	74	1.00		106	1.00	
TG	49	15	1.03 (0.59 – 1.80)	0.912	19	0.91 (0.56 – 1.48)	0.703
GG	3	0	–	0.987	0	–	0.989
Additive model			1.02 (0.59 – 1.78)	0.937		0.91 (0.56 – 1.48)	0.697
Dominant model	52	15	1.03 (0.59 – 1.79)	0.923	19	0.91 (0.56 – 1.48)	0.700
Recessive model	3	0	–	0.987	0	–	0.989
XPC							
rs2228001							
AA	129	34	1.00		47	1.00	
AC	152	48	1.24 (0.79 – 1.93)	0.345	67	1.32 (0.91 – 1.94)	0.148
CC	36	6	0.76 (0.32 – 1.81)	0.532	9	0.74 (0.36 – 1.52)	0.417
Additive model			1.01 (0.73 – 1.40)	0.952		1.02 (0.78 – 1.33)	0.900
Dominant model	188	54	1.16 (0.75 – 1.79)	0.513	114	1.21 (0.84 – 1.75)	0.312
Recessive model	36	6	0.67 (0.29 – 1.54)	0.347	9	0.64 (0.32 – 1.26)	0.192
rs2228000							
CC	137	34	1.00		58	1.00	
TC	147	42	1.01 (0.64 – 1.59)	0.984	53	0.79 (0.54 – 1.15)	0.214
TT	35	13	1.61 (0.85 – 3.05)	0.145	14	0.99 (0.53 – 1.85)	0.984
Additive model			1.19 (0.87 – 1.65)	0.277		0.91 (0.68 – 1.20)	0.493
Dominant model	182	55	1.11 (0.72 – 1.70)	0.644	67	0.82 (0.58 – 1.17)	0.276
Recessive model	35	13	1.60 (0.89 – 2.89)	0.117	14	1.12 (0.62 – 2.03)	0.712

XPA							
rs1800975							
AA	83	26	1.00		31	1.00	
AG	164	45	0.89 (0.55 – 1.44)	0.630	68	1.07 (0.70 – 1.65)	0.744
GG	72	18	0.82 (0.45 – 1.50)	0.521	26	0.95 (0.57 – 1.60)	0.851
Additive model			0.91 (0.67 – 1.22)	0.512		0.98 (0.76 – 1.26)	0.873
Dominant model	236	63	0.87 (0.55 – 1.37)	0.542	94	1.04 (0.69 – 1.56)	0.867
Recessive model	72	18	0.89 (0.53 – 1.49)	0.649	26	0.91 (0.59 – 1.40)	0.658
rs10817938							
TT	204	56	1.00		74	1.00	
TC	105	28	1.03 (0.65 – 1.63)	0.897	46	1.26 (0.87 – 1.83)	0.226
CC	7	5	3.00 (1.19 – 7.52)	0.020*	5	2.83 (1.14 – 7.04)	0.025*
Additive model			1.27 (0.87 – 1.85)	0.225		1.39 (1.00 – 1.92)	0.048*
Dominant model	112	33	1.14 (0.74 – 1.76)	0.544	51	1.34 (0.93 – 1.92)	0.118
Recessive model	7	5	2.97 (1.20 – 7.35)	0.019*	5	2.61 (1.06 – 6.41)	0.037*

OSCC, Oral squamous cell carcinoma; SNPs, single nucleotide polymorphisms; HR, hazard ratio; CI, confidence interval.

* $p<0.05$

Table S3. Haplotype analysis of association between NER candidate SNPs and OSCC survival in patients treated with CCRT

		Overall survival		Disease-free survival	
Haplotypes	Frequency (%)	HR (95% CI)[a]	*p* value	HR (95% CI)[b]	*p* value
ERCC5 block1[c]					
GT	37.62	1.00		1.00	
GC	27.74	0.84(0.58–1.21)	0.344	0.86(0.65–1.21)	0.447
AT	34.48	0.84(0.59–1.20)	0.330	0.98(0.73–1.33)	0.913
AC	0.16	-	0.985	-	0.977
ERCC5 block2[d]					
GA	52.20	1.00		1.00	
GG	2.51	0.48 (0.15–1.51)	0.208	0.81 (0.38–1.75)	0.595
CA	0.31	-	0.982	-	0.977
CG	44.98	0.93 (0.69–1.26)	0.648	1.15 (0.89–1.49)	0.271
ERCC1[e]					
GC	39.97	1.00		1.00	
TC	33.07	1.14(0.80–1.64)	0.471	0.90(0.67–1.21)	0.471
GT	26.96	1.15(0.79–1.66)	0.467	0.82(0.60–1.12)	0.216
XPC[f]					
AC	30.56	1.00		1.00	
CC	35.42	1.12 (0.77–1.64)	0.557	0.96 (0.71–1.31)	0.813
AT	34.01	1.31 (0.90–1.89)	0.159	0.93 (0.68–1.27)	0.633
XPA[g]					
AT	51.72	1.00		1.00	
GT	29.62	0.68 (0.47–0.99)	0.042*	0.75 (0.56–1.02)	0.066
GC	18.65	1.05 (0.72–1.54)	0.796	1.19 (0.86–1.64)	0.295

OSCC, oral squamous cell carcinoma, SNPs, single nucleotide polymorphisms; HR, hazard ratio; CI, confidence interval.

[a] Adjusted for age, BMI, N stage, lymphatic invasion, and extranodal extension.

[b] Adjusted for age, T stage, N stage, and extranodal extension.

[c] Haplotypes of rs2094258 and rs1047768.

[d] Haplotypes of rs17655 and rs873601.

[e] Haplotypes of rs3212986 and rs11615.

[f] Haplotypes of rs2228001 and rs2228000.

[g] Haplotypes of rs1800975 and rs10817938.

* $p < 0.05$
